# Predictors of residual low back pain in patients with osteoporotic vertebral fractures following percutaneous kyphoplasty

**DOI:** 10.3389/fsurg.2023.1119393

**Published:** 2023-02-03

**Authors:** Hongwei Yu, Gan Luo, Ziqi Wang, Bin Yu, Tianwei Sun, Qiong Tang

**Affiliations:** ^1^ School of Medicine, Nankai University, Tianjin, China; ^2^ Graduate School of Tianjin Medical University, Tianjin, China; ^3^ Department of Spinal Surgery, Tian-jin Union Medical Centre, Nankai University People's Hospital, Tianjin, China; ^4^ Department of Respiratory Medicine, Tian-jin Union Medical Centre, Nankai University People's Hospital, Tianjin, China

**Keywords:** low back pain, osteoporotic vertebral fractures, percutaneous kyphoplasty, risk factors, nomogram

## Abstract

**Objective:**

Patients with osteoporotic vertebral fractures (OVFs) often suffer from residual low back pain (LBP) after percutaneous kyphoplasty (PKP). The purpose of this study was to identify risk factors for postoperative residual LBP and to develop a nomogram to predict the occurrence of residual LBP.

**Methods:**

We retrospectively reviewed 236 patients who underwent PKP for OVFs and had a minimum follow-up of 12 months. The mean age was 72.1 ± 6.3, 74.3% were female and 25.7% were male. Patients with LBP VAS scores ≥ 3.5 at the 12th month postoperatively were considered to have residual LBP. Risk factors for residual LBP were identified by univariate and multifactorial logistic regression analysis. Then, a predictive nomogram was constructed and validated using the bootstrap method. The discrimination, calibration, and clinical utility of the nomogram were assessed using a receiver operating characteristic curve (ROC), a calibration curve, and a decision curve analysis (DCA).

**Results:**

univariate and multifactorial logistic regression analysis identified depression (*P* = 0.02), intravertebral vacuum cleft (*P* = 0.01), no anti-osteoporosis treatment (*P* < 0.001), cement volume <3 ml (*P* = 0.02), and cement distrubution (*P* = 0.01) as independent risk factors for residual LBP. The area under the ROC was 0.83 (0.74–0.93) and further validated by bootstrap method was 0.83 (0.73–0.92). The calibration curve illustrated the consistency between the predicted probability and the observed results. DCA showed that nomogram exhibits clinical utility and net benefit when the threshold probability is between 6% and 73%.

**Conclusions:**

Our study found that depression, intravertebral vacuum cleft, no anti-osteoporosis treatment, cement volume <3 ml and cement distribution represent independent risk factors for residual LBP. The nomogram containing the above five predictors can accurately predict the risk of residual LBP after surgery.

## Introduction

Osteoporotic vertebral fractures (OVFs) result from decreased bone density and bone strength (1). OVFs mainly cause chronic and persistent low back pain, secondary kyphosis, and even cardiopulmonary complications, which seriously affect the quality of life of middle-aged and older adults (2, 3). Percutaneous balloon kyphoplasty (PKP) is performed by expanding the compressed vertebral body with a balloon and injecting polymethyl methacrylate (PMMA) bone cement into the expanded cavity. This method can provide pain alleviation and kyphosis correction and is considered to be the current preferred minimally invasive surgical treatment (4). However, some patients still have persistent moderate to severe low back pain after PKP with an incidence ranging from 1.8% to 15.6% (5, 6). The presence of postoperative residual low back pain may weaken the outcomes of the PKP procedure, decrease the patients' quality of life and increase their financial burden. Therefore, it is crucial to explore the baseline risk factors for residual LBP in patients with OVFs following PKP.

Studies have examined various potential risk factors for residual LBP, such as bone mineral density, intravertebral vacuum cleft, posterior fascia edema, fracture of adjacent vertebral bodies, and vertebral compression ratio (5, 7, 8). However, preoperative mental status and intraoperative factors, such as cement volume and distribution, were not analyzed as risk factors (9, 10). More importantly, shorter follow-up periods (1 month) and higher pain thresholds (VAS ≥ 4) may not accurately identify patients with residual LBP (10) because we cannot be certain whether early postoperative residual pain will be resolved spontaneously during long-term follow-up. Therefore, a predictive model based on long-term follow-up and including mental status and intraoperative factors should be developed to guide therapeutic interventions for residual LBP.

Our study analyzed multiple baseline factors associated with residual LBP after PKP in OVFs. Furthermore, we developed and validated a nomogram to provide individualized guidance for the treatment of residual LBP.

## Methods

### Patients

**Figure 1:**
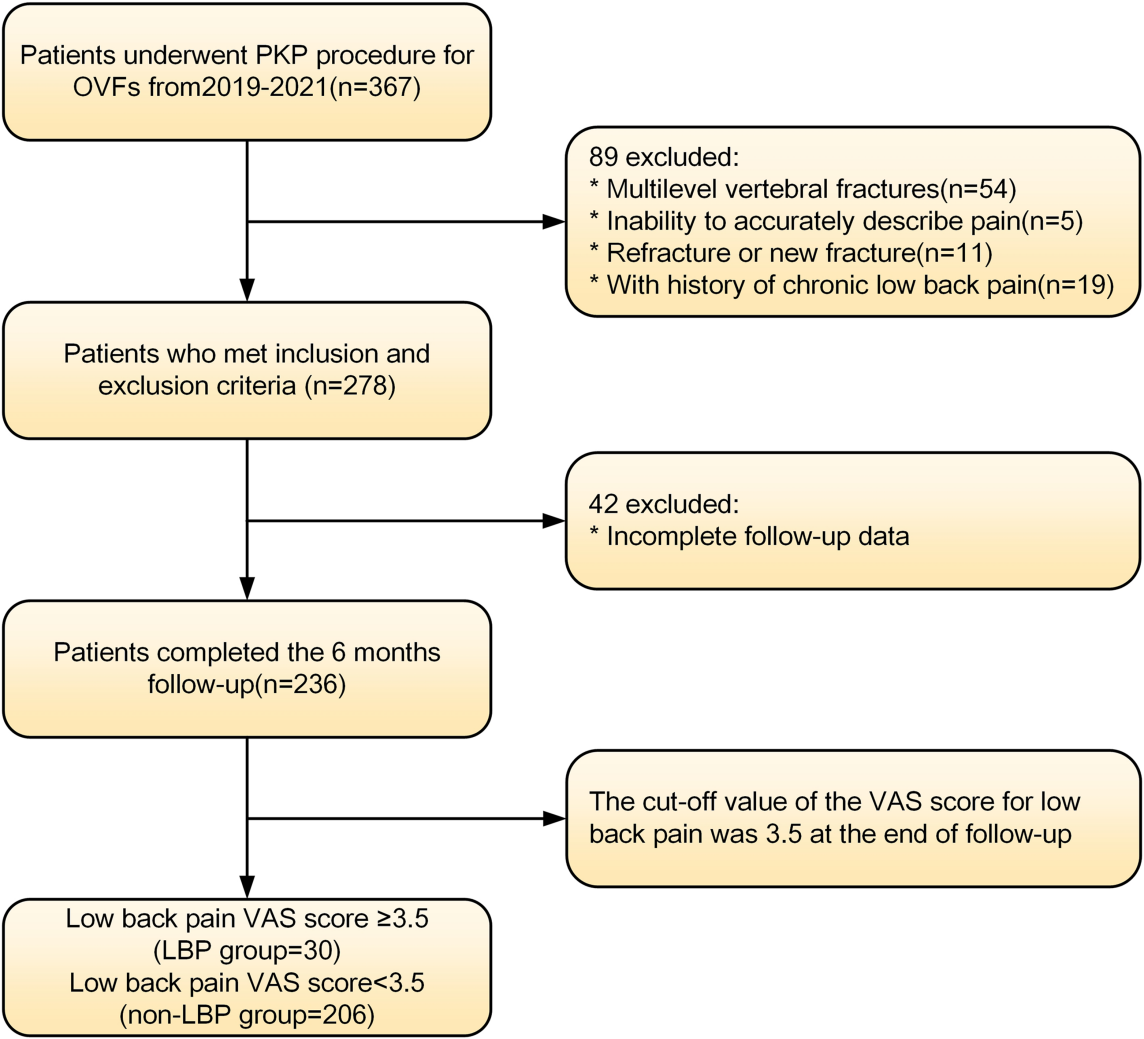
Patients selection flowchart.

We prospectively collected data from patients treated with PKP for osteoporotic vertebral fractures between May 2019 and June 2021 at a hospital in Tianjin, China. These patients were analyzed retrospectively. The details of the inclusion and exclusion criteria were as follows. Inclusion criteria: (1) The patient had a clear history of osteoporosis or was diagnosed with osteoporosis by DXA. (2) Vertebral fractures caused by low-energy injuries and further confirmed by spinal MRI (11). (3) Only patients with single-segment vertebral fractures were included in this study. (4) The patients had obvious low back pain symptoms because of the vertebral fractures (VAS > 3.5). (5) Patients were treated with PKP rather than conservative or other treatments. (6) Information on the clinical and demographic characteristics required for the study was complete and accessible. The exclusion criteria were as follows: (1) Pathological fractures caused by tumors, infections, tuberculosis or other diseases. (2) Spinal cord compression and obvious neural symptoms, such as numbness and/or muscle weakness. (3) Patients had chronic low back pain(VAS > 3.5) prior to the fractures, either due to osteoporosis, degenerative kyphosis or scoliosis, idiopathic pain, previous back surgery, or other diseases. (4) Patients with cognitive disorders who could not communicate independently. Figure 1 shows the screening process for enrolling patients. This study was approved by the Medical Ethics Committee of the Tianjin Union Medical Centre and conducted in accordance with the Declaration of Helsinki. Informed consent was waived because this is a retrospective study that does not involve personal privacy or commercial interests.

### Surgical technique

The patient was placed in a prone position, raised to make the abdomen hang, and routinely sterilized. The C-arm machine was used to locate the fractured vertebrae following local anesthesia or general anesthesia. Using pedicle approach, the puncture probe was inserted into the vertebrae. The tip in the lateral fluoroscopy was located in the front 1/3 of the vertebral body and close to or exceeded the midline of the spinous process in the front fluoroscopy. Then, the tube core of the guide needle was removed, and the working channel was established. The inflatable balloon was placed into the working channel, and the contrast medium was slowly injected into the balloon. The balloon was expanded to reposition the vertebral body. The vertebral body was filled with bone cement under continuous fluoroscopic guidance to eliminate the expansion gap. The procedure should be stopped immediately in cases with bone cement leakage. After curing the bone cement, the working channel was withdrawn. Then, the skin incision was sutured, and the cells were observed for 10–20 min. Radiographs and computed tomography (CT) scans of the spine were taken for all patients on the first postsurgical day. All patients were discharged 2 to 3 days after surgery.

### Identification of residual low back pain

A visual analog scale was used to evaluate patients' low back pain intensity at the 12th month. Patients' low back pain intensity is the average intensity for the month. A VAS score <3.5 corresponds to mild pain, whereas a VAS score ≥3.5 corresponds to moderate to severe pain and is considered to indicate residual low back pain (12). Spine MRI should be performed at any time to exclude the suspicion of a refracture or a new fracture at another level.

### Data collection

Baseline risk factors were extracted from the medical records, operative records, radiological image management system, and questionnaire surveys. Two independent spinal surgeons were involved in the radiographic evaluation. When a disagreement occurred between the surgeons, a consensus meeting was held.

Baseline demographic characteristics included age, sex, body mass index (BMI), bone mineral density (BMD), comorbidities [hypertension, diabetes, and chronic obstructive pulmonary disease (COPD)], smoking, and depression. The depression status of patients was determined based on the mental component summary of the Short Form-36 (SF-36 MCS) (13). SF-36 MCS > 50 indicates depression; otherwise, depression is not noted.

**Figure 2:**
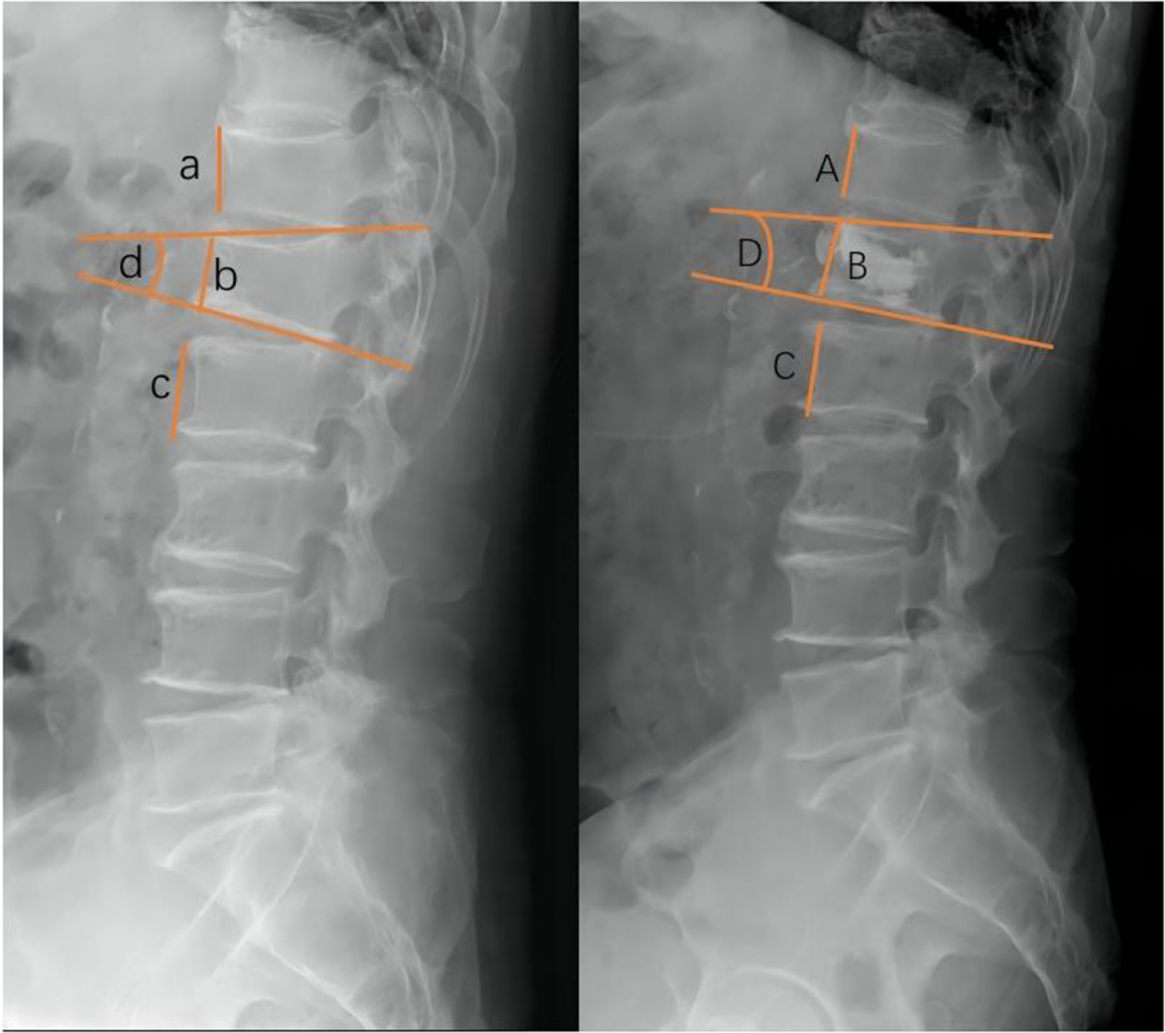
Radiographic evaluation of compressed vertebrae. Preoperative VHR (left,%):2b/a + c, VKA (left,°):∠d; Postoperative VHR (right,%):2B/A + C, VKA (right,°):∠D.

**Figure 3:**
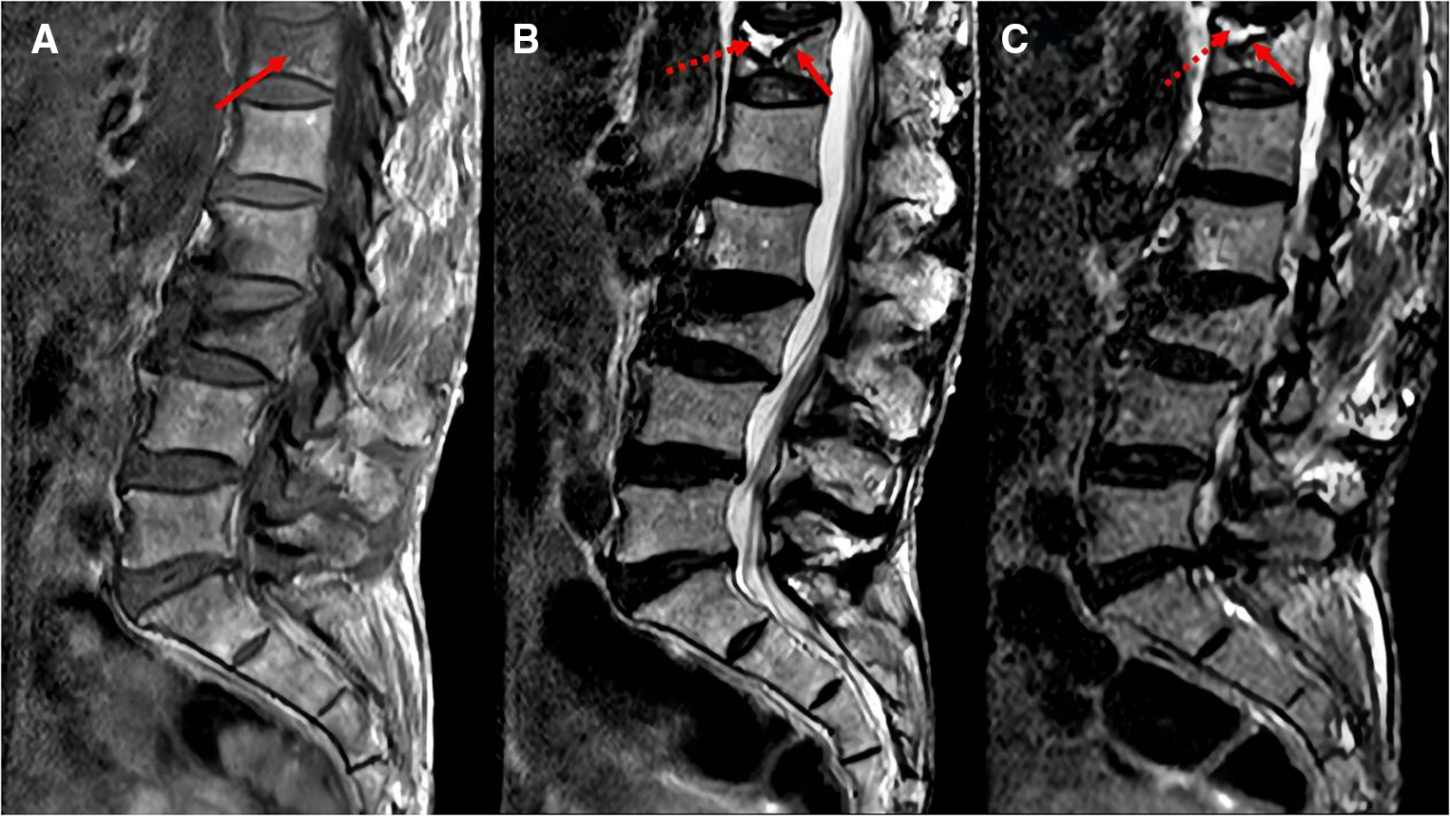
MRI images of intravertebral vacuum cleft. Hypointense on T1-weighted images (**A**, arrows); hyperintensity or hypointensity on T2-weighted images (**B**) or STIR images (**C**), depending on fluid (dotted arrows) or gas (solid arrows) filling.

**Figure 4:**
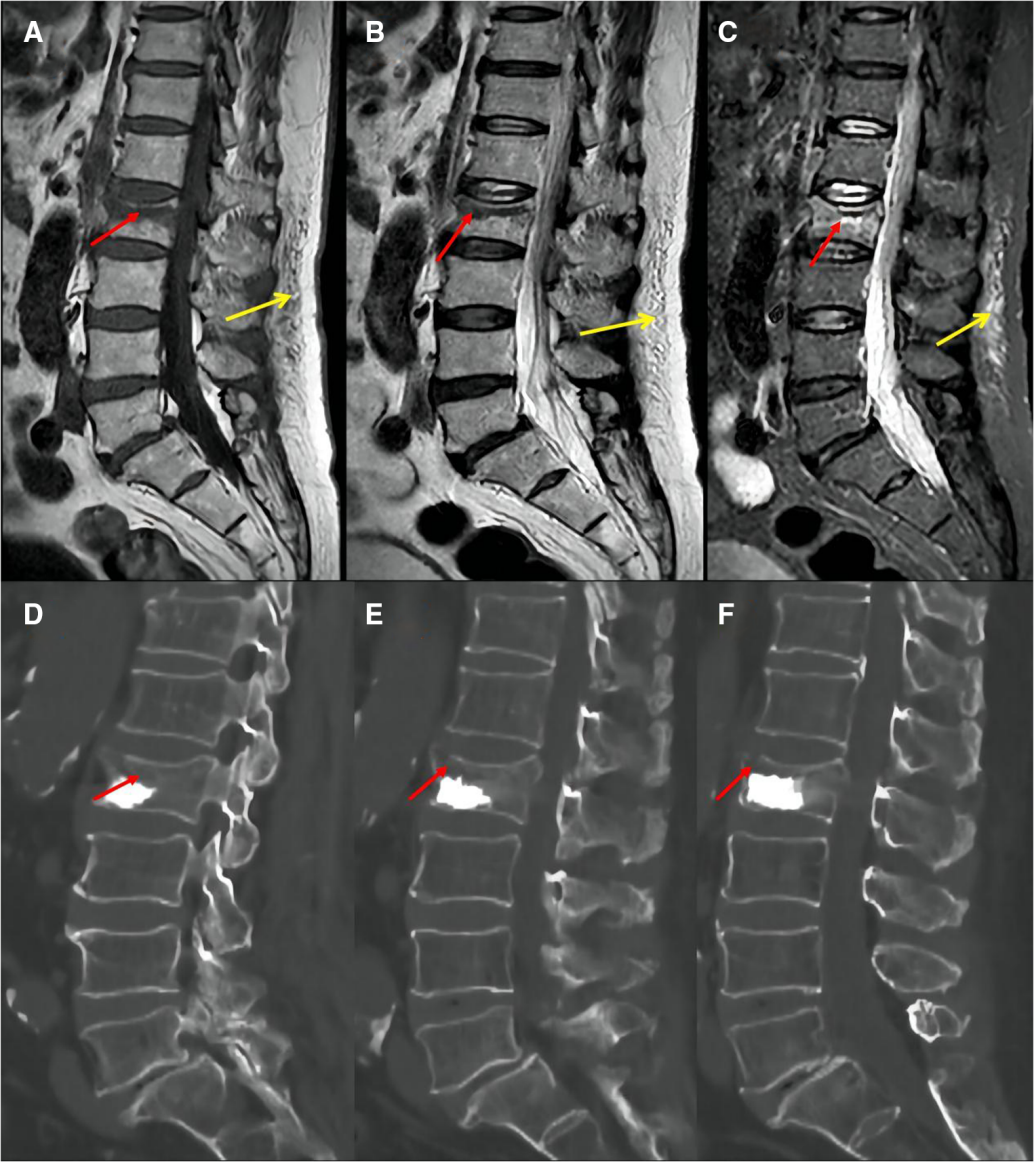
Preoperative MRI images showed that the fracture area was located in the upper third of the vertebral body (**A**: T1, **B**: T2, **C**: STIR, red arrow); postoperative CT images showed insufficient cement filling in the vertebral fractured area (**D**: left in median sagittal section; **E**: median sagittal section; **F**: right in median sagittal section, red arrow). Posterior fascia edema:hypointense on T1-weighted images (**A**, yellow arrow); hyperintensity on T2-weighted images (**B**, yellow arrow) or STIR images (**C**, yellow arrow).

**Figure 5:**
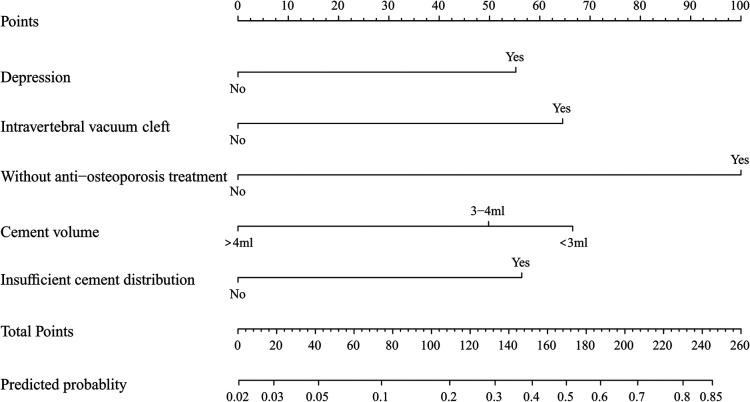
Predictive nomogram for residual LBP.

The following baseline clinical and radiological factors were assessed: (1) Time from pain to surgery; (2) Segment location—thoracic spine (T4–T9), thoracolumbar spine (T10-L2), and lumbar spine (L3–L5); (3) VAS score for low back pain; (4) Vertebral height ratio (VHR) and vertebral kyphosis angle (VKA)—The vertebral height ratio is the ratio of the anterior height of the fractured vertebral body to the average anterior height of the adjacent upper and lower segments (Figure 2), and the vertebral kyphosis angle is the angle between the upper and lower endplates of a fractured vertebral body (Figure 2); (5) Anti-osteoporosis therapy—there are two anti-osteoporosis protocols, intravenous zoledronic acid (5 mg/year) or oral alendronate sodium (70 mg/week). Given compliance issues with alendronate administration, we considered patients who had been taking alendronate for less than 6 months as not receiving regular anti-osteoporosis treatment; (6) Vitamin D (800–1,200 IU/d) and calcium supplements (1000 mg/d)—Given issues with medication adherence, we considered patients who did not receive or those who received vitamin D and calcium for less than 6 months as not receiving a standardized anti-osteoporosis basal supplement. (7) Intravertebral vacuum cleft (IVC; Figure 3); (8) Posterior fascia edema (Figure 4).

Intraoperative factors: (1) Anesthesia—local or general anesthesia; (2) Cement volume; (3) Cement distribution—Cement distribution was classified into two states of sufficient and insufficient based on whether the cement filled the majority of the fractured area of the vertebrae (Figure 4); (4) Cement leakage.

### Statistical analysis

Univariate analysis was conducted to determine whether these factors were associated with residual LBP. The normal distribution of continuous data was assessed using the Shapiro‒Wilk test, and the between-group differences were evaluated using the Student's t test or the Mann‒Whitney *U* test according to the data distribution characteristics. Chi-squared or Fisher's exact test was used to investigate differences in categorical data between the two groups. Variables with *P* value less than 0.05 in the univariate analysis were included in further multifactorial analyses to identify independent risk factors. The nomogram was then developed using independent risk factors. SPSS 26 software (IBM, USA) was used for data analysis, and R software (version 4.1.1, Foundation for Statistical Computing, Vienna, Austria) was utilized for nomogram construction. The predictive ability and performance of the model were assessed based on the receiver operating characteristic (ROC) curve, the area under the ROC curve (AUC), the calibration curve, and decision curve analysis (DCA). In general, the AUC values between 0.5 and 1.0 indicate that the model can accurately predict and discriminate (14). The calibration curve was used to assess the consistency between the actual diagnosis of LBP and the projected likelihood of LBP. Furthermore, by estimating the net benefit and threshold probabilities of the nomogram, DCA was used to evaluate its usefulness in clinical practice. Finally, we performed internal validation using 1,000 bootstrap samples to assess the stability of the prognostic nomogram.

## Results

A total of 236 patients were enrolled in this study and completed the 12-month follow-up according to the inclusion and exclusion criteria. Thirty patients were included in the LBP group (VAS ≥ 3.5), and the incidence of residual LBP following PKP was 12.7%. Another 206 patients with a low back pain VAS score <3.5 were included as the non-LBP group. Both groups experienced significant improvement in VAS scores compared with the preoperative scores at the end of follow-up (*P* < 0.01), but significant differences existed between the two groups (*P* < 0.01), as shown in Table 1.

**Table 1:** Low back pain VAS results at the 12th month.

Characteristic	VAS < 3.5 (*n* = 206)	VAS ≥ 3.5 (*n* = 30)	*P* Value
Preoperative	7.1 ± 0.6	7.2 ± 0.4	0.54
1 day after surgery	4.9 ± 0.7	5.0 ± 0.6	0.17
12th month	2.2 ± 0.6	3.8 ± 0.4	<0.01
*P* Value*	<0.01	<0.01	

* Mean low back pain VAS score at month 12 postoperatively compared to preoperatively.

### Univariate and multivariate analysis

Univariate analysis results of baseline demographic characteristics, baseline clinical and radiological factors, and intraoperative factors are shown in Tables 2. The two groups differed significantly in terms of depression (*P* = 0.01), intravertebral vacuum cleft (*P* < 0.01), anti-osteoporosis treatment (*P* < 0.01), cement volume (*P* = 0.01), and cement distribution (*P* = 0.01). None of the other variables differed significantly between the two groups. Multivariate analysis indicates that depression (OR = 3.56, 95% CI: 1.19–10.64, *P* = 0.02), intravertebral vacuum cleft (OR = 4.41, 95% CI: 1.57–12.36, *P* = 0.01), no anti-osteoporosis treatment (OR = 9.63, 95% CI: 3.50–28.33, *P* < 0.01), insufficient cement distribution in the fractured area (OR = 3.66, 95% CI: 1.36–9.86, *P* = 0.01), and cement volume <3 ml (OR = 4.62, 95% CI: 1.26–16.91, *P* = 0.02) were independent risk factors for residual LBP, as shown in Table 3.

**Table 2:** Demographic, clinical and radiological characteristics of the patients collected.

Variables	VAS < 3.5	VAS ≥ 3.5	*X*^2^/t	*P*-Value	Power calculation
Age (years)			1.27	0.26	0.07
≤75	157 (76.2)	20 (66.7)			
>75	49 (23.8)	10 (33.3)			
Sex			0.55	0.46	0.05
Female	167 (81.1)	26 (86.7)			
Male	39 (18.9)	4 (13.3)			
BMI (kg/m2)			0.80	0.31	0.06
<24	72(35)	8 (26.7)			
≥24	134 (65)	22 (73.3)			
BMD (T score)			2.51	0.21	0.10
<−2.5	53 (25.7)	11 (36.7)			
≥−2.5	153 (74.3)	19 (63.3)			
Hypertension			1.92	0.17	0.09
Yes	82 (39.8)	8 (26.7)			
No	124 (60.2)	22 (73.3)			
Diabetes			0.02	0.89	0.01
Yes	39 (18.9)	6 (20)			
No	167 (81.1)	24 (80)			
COPD			1.01	0.31	0.07
Yes	11 (5.3)	3 (10)			
No	195 (94.7)	27 (90)			
Smoking			1.17	0.31	0.07
Yes	27 (13.1)	6 (20)			
No	179 (86.9)	24 (80)			
Depression			6.10	0.01*	0.16
Yes	31 (15)	10 (33.3)			
No	175 (85)	20 (66.7)			
Time from pain to surgery			1.46	0.23	0.08
<6 weeks	170 (82.5)	22 (73.3)			
≥6 weeks	36 (17.5)	8 (26.7)			
Segments location			0.47	0.79	0.04
T	14 (6.8)	2 (6.7)			
TL	170 (82.5)	26 (86.7)			
L	22 (10.7)	2 (6.7)			
Intravertebral vacuum cleft			14.12	**<0**.01*	0.24
Yes	19 (9.2)	10 (33.3)			
No	187 (90.8)	20 (66.7)			
Posterior fascia oedema			1.04	0.31	0.07
Yes	27	6 (20)			
No	179	24 (80)			
Preoperative					
VAS	7.2 ± 0.7	7.5 ± 1.9	−0.61	0.54	0.32
VHR (%)	63.2 ± 8.5	61.9 ± 7.9	0.79	0.43	0.26
VKA (°)	24.0 ± 7.0	25.8 ± 7.1	−1.37	0.17	0.02
1 day after surgery					
VAS	4.9 ± 0.6	5.0 ± 0.6	−1.37	0.16	0.02
VHR (%)	78.6 ± 8.9	76.7 ± 8.4	1.12	0.26	0.11
VKA (°)	11.8 ± 7.5	13.6 ± 7.6	−1.23	0.22	0.04
Anti-osteoporosis			15.32	<**0**.01*	0.25
Yes	177 (85.9)	17 (56.7)			
No	29 (14.1)	13 (43.3)			
Vitamin D and calcium			2.19	0.13	0.09
Yes	189 (91.7)	25 (83.3)			
No	17 (8.3)	5 (16.7)			
Anesthesia			0.31	0.58	0.04
Local	197 (95.6)	28 (93.3)			
General	9 (4.4)	2 (6.7)			
Cement volume (ml)			9.80	**0**.01*	0.20
>4	172 (83.5)	19 (63.3)			
3–4	23 (11.2)	5 (16.7)			
<3	11 (5.3)	6 (20)			
Cement distribution			6.86	**0**.01*	0.17
Sufficient	167 (81.1)	18 (60)			
Insufficient	39 (18.9)	12 (40)			
Cement leakage			0.09	0.76	0.02
Yes	30 (14.6)	5 (16.7)			
No	176 (85.4)	25 (83.3)			

Continuous variables are presented as mean ± standard deviation and categorical variables are presented as number (%).

*Results are statistically significant.

**Table 3:** Multivariate logistic regression analysis of independent risk factors for residual low back pain.

Risk factors	Odds ratio	95% CI	*P* value
Depression	3.56	1.19–10.64	0.02
Intravertebral vacuum cleft	4.41	1.57–12.36	0.01
No anti-osteoporosis treatment	9.96	3.50–28.33	<0.01
Cement volume			0.03
3–4 ml vs. >4 ml	3.14	0.85–11.67	0.09
<3 ml vs. >4 ml	4.61	1.26–16.91	0.02
Insufficient cement distribution	3.66	1.36–9.86	0.01

### Development and validation of a nomogram for residual LBP

**Figure 6:**
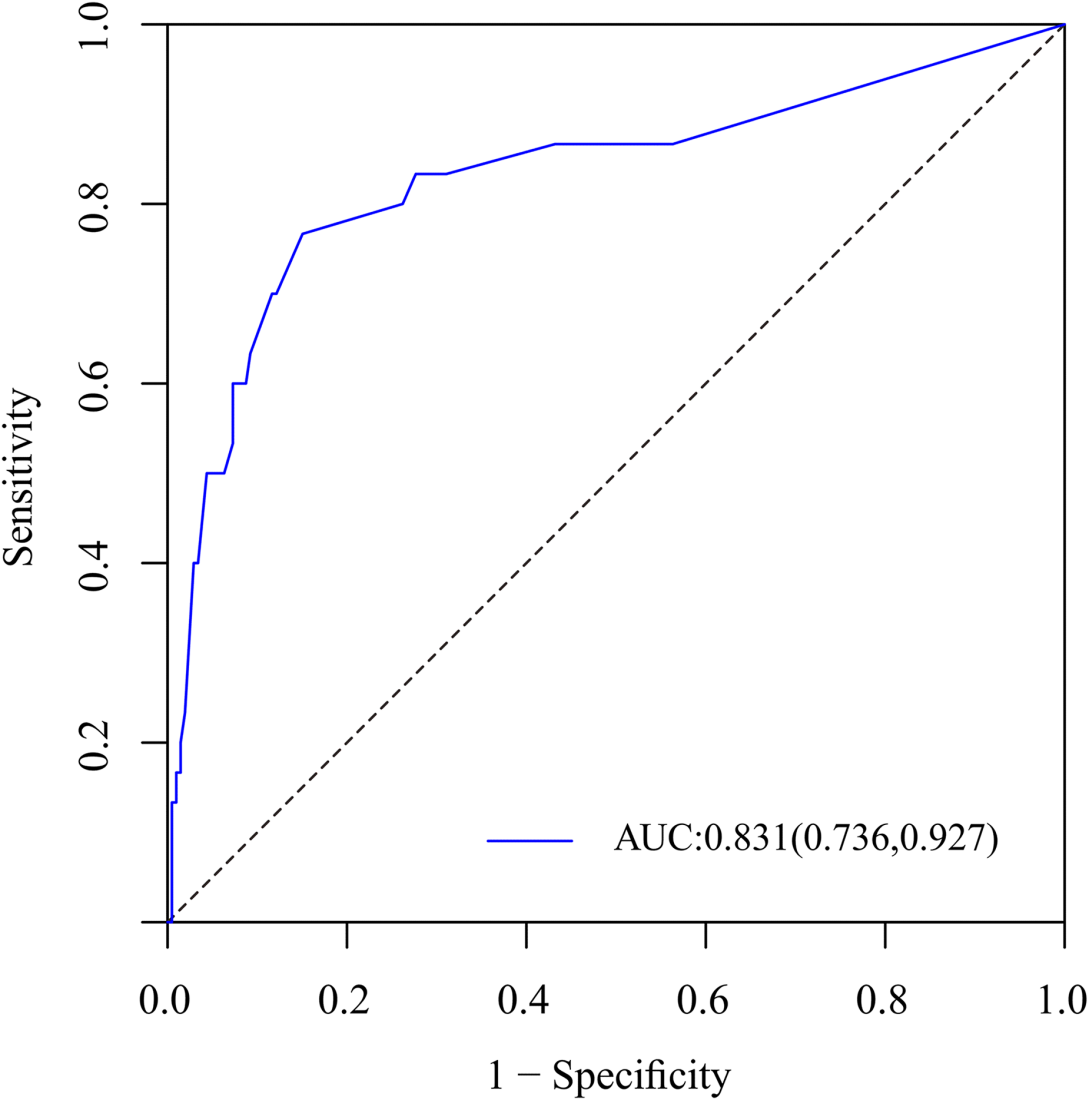
ROC curves of the nomogram for the assessment of capable of accurate prediction and discrimination.

**Figure 7:**
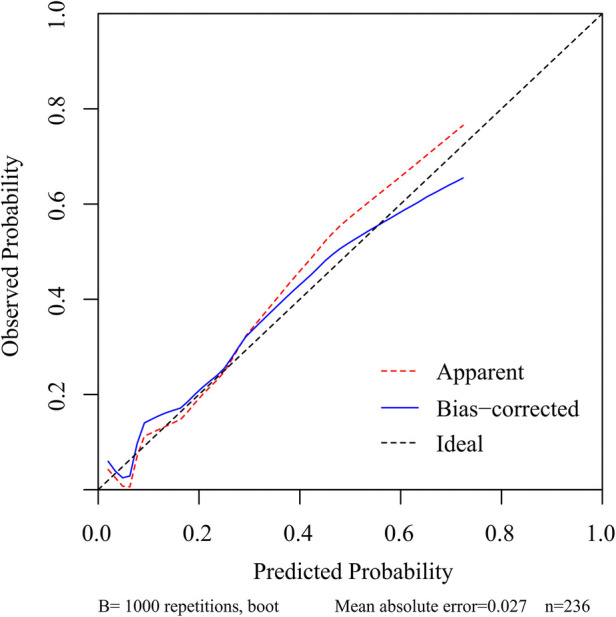
Calibration curve of the nomogram for the assessment of the consistency between the actual diagnosed residual LBP and the projected likelihood of residual LBP.

**Figure 8:**
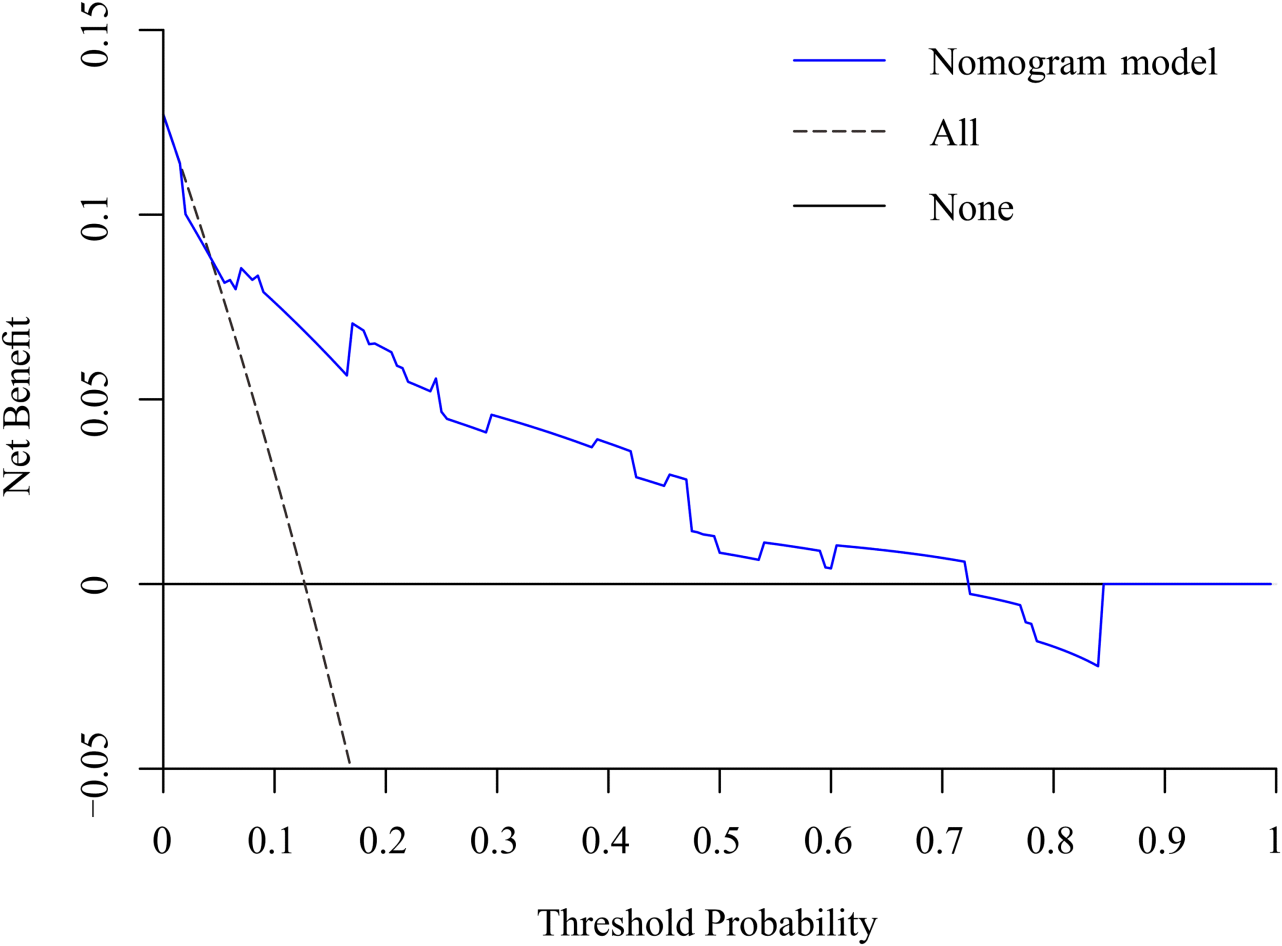
DCA curve of the nomogram for the assessment of clinical practice based on the net benefit and threshold probabilities.

To predict residual LBP, we developed a nomogram using the predictive factors identified in multivariate analysis (Figure 5). The predictive score of each factor was added together in the nomogram to calculate the total score, from which the probability of residual LBP occurrence was calculated (Figure 5). The model has high predictive accuracy and discrimination with an AUC of 0.83 (0.74–0.93) and a corrected AUC of 0.83 (0.73–0.92) based on bootstrapping validation, as shown in Figure 6. Furthermore, the calibration curve of the nomogram demonstrated an excellent consistency between the actual diagnosis of residual LBP and the predicted likelihood (Figure 7). Similarly, the DCA curves indicate that the nomogram would provide a high net benefit when applied to the clinic (Figure 8).

## Discussion

In this study, we analyzed 236 patients with osteoporotic vertebral fractures to identify baseline impact factors for postoperative residual LBP and developed and validated a predictive model to predict the risk of LBP occurrence following PKP. We found that 12.7% of patients experienced residual LBP postoperatively, which is similar to that reported in previous studies (5, 9). Multifactorial logistic regression analysis identified depression, intravertebral vacuum cleft, no anti-osteoporosis treatment, insufficient cement distribution in the fractured area, and cement volume <3 ml as key predictive factors related to residual LBP. The above factors were included in the nomogram.

Several studies have shown that depression can affect pain and function following surgery for musculoskeletal disorders (15, 16). The Mental Component Score (MCS) of the Short Form-36 (SF-36) has been used in numerous studies as an indicator of depression or depressive tendencies to predict surgical outcomes for lumbar degenerative diseases (17–19). It was found that patients with depressive symptoms based on their MCS experienced worse performance postoperatively in health-care-related quality of life (HRQOL) than patients without depression (18). Kimura et al. (13) found that the presence of depression (SF-36 MCS < 50) was associated with persistent neck pain following cervical laminoplasty and significantly worse functional outcomes. According to this study, depressed patients may experience residual low back pain after PKP. These results suggest that the presence of depression affects not only clinical outcomes following spinal fusion surgery but also those following percutaneous kyphoplasty.

The pathogenesis of the intravertebral vacuum cleft is unclear. Most studies (20, 21) tend to indicate avascular necrosis of trabecular bone. On T2-weighted or STIR images, IVC typically showed high or low signal intensity, depending on whether fluid or gas fills the cleft (22, 23). Many scholars agree that a strong correlation exists between IVC and residual low back pain, vertebral recollapse, and instability (24). A retrospective study of at least two years (25) found that low back pain VAS scores in patients with IVC were higher than those without IVC, and the height loss of the vertebral body in the IVC group was greater than that noted in the non-IVC group at the final follow-up. A meta-analysis by Yu et al. (26) also found that improvement in VAS back pain scores was limited in patients with IVC. Similar to these earlier studies, our results revealed that IVC is a vital contributor to residual low back pain.

The pathological basis of OVCF is osteoporosis, and PKP can help patients relieve pain and regain self-care early. However, the procedure only treats fractured vertebrae and has no therapeutic effect on systemic osteoporosis. Previous reports (27) have noted a 19.2% incidence of new vertebral fractures in the year following treatment in those who had vertebral fractures. Several studies (28, 29) have found that the application of bisphosphonate anti-osteoporosis therapy after PKP reduced vertebral refractures or new fractures and reduced low back pain VAS scores. During the follow-up period of our study, we identified refractures or new fractures using spinal MR images in combination with physical examination, but we lacked an effective means to identify microfractures of the vertebral body. More importantly, because patients can tolerate this low level of chronic low back pain, they do not go to the hospital for treatment. Several studies (30, 31) have shown that postoperative anti-osteoporotic treatment can prevent height loss after vertebral PKP, and we believe that this finding may serve as indirect evidence indicating that anti-osteoporotic treatment can reduce vertebral microfractures because the most visual manifestation of microfractures is vertebral height loss. Therefore, standardized anti-osteoporosis drug therapy should be actively taken after surgery to relieve pain, inhibit acute bone loss, increase bone strength, improve bone quality, and reduce fractures or new fractures.

Cement volume and cement distribution in the fractured area are independent predictors of residual low back pain that may be related to the inadequate restoration of vertebral stiffness and strength (32). With at least 6 months of follow-up, Christoph (33) found only a 40% chance of attaining a minimum 41-point pain alleviation when the cement volumes were less than 4.5 ml, and the most suitable fill volume for PKP seems to be between 4.5–7.5 ml. Fu et al. (34) demonstrated that cement dosage is positively correlated with pain alleviation. Therefore, the authors recommended that the vertebral body should be injected with as much cement as possible when performing the PKP procedure. More importantly, the cement volume can be changed intraoperatively. Sufficient bone cement diffusion in the fractured area after PKP stabilizes micromotion in the fracture area, limiting local nerve ending stimulation and relieving fracture-induced pain (35, 36). Furthermore, we hypothesize that inadequate cement volume and insufficient distribution of cement in the fractured area were associated with vertebral deformity in the coming later. Although there was no significant difference in vertebral body height or kyphosis angle between the LBP and non-LBP groups measured 1 day after surgery, inadequate recovery of vertebral body strength and stiffness resulting from inadequate cement volume or insufficient distribution in the fracture area may result in progressive vertebral kyphosis deformity over time. Multiple studies have demonstrated that the presence of vertebral kyphosis is a critical risk factor for long-term persistent low back pain after osteoporotic vertebral fractures (37, 38). Future prospective studies are needed to clarify the relationship between cement volume, cement distribution, and progressive vertebral kyphosis following PKP.

After analyzing potential risk factors, such as demographic characteristics, clinical and radiological factors, and intraoperative factors, we developed a nomogram for predicting residual LBP. Intraoperative interventions can be performed to modify these risk factors in patients, such as the amount and distribution of cement. Additionally, this study emphasizes the need for standardized anti-osteoporosis treatment for OVCF patients after PKP.

Our research also has some limitations. (1). Although we collected mean VAS scores for low back pain in patients at the 12th month postoperatively, it is still possible that this low back pain is incidental; therefore, studies with long follow-up periods and multiple follow-up nodes are needed to further validate the findings. (2). Patients have different ranges of pain tolerance, leading to overestimating or underestimating the residual LBP rate. (3). As this was a retrospective study, some confounding factors were unavoidable, such as the patient's preoperative low back muscle mass, daily activities that may contribute to the sensation of low back pain, comorbidity with other underlying conditions, differences in philosophy and technique between surgeons, and the patient's socioeconomic status. (4). We exclusively administered bisphosphonates for anti-osteoporosis treatment, and the effects anti-osteoporosis drugs with different mechanisms of action (e.g., denosumab, teriparatide, estrogen replacement therapy) on postoperative residual low back pain remain unclear. (5). This study is a single-center study from China, and future multicenter studies in different hospitals, regions, or countries are needed to further validate the findings of this study.

## Conclusion

Our study found that depression, intravertebral vacuum cleft, no anti-osteoporosis treatment, insufficient cement distribution in the fractured area, and cement volume <3 ml are independent risk factors for residual LBP after PKP treated for OVFs. The nomogram containing the above five predictors can accurately predict the risk of residual LBP after surgery.

## Data Availability

The original contributions presented in the study are included in the article/Supplementary Material, further inquiries can be directed to the corresponding author/s.
